# Elucidation and functional characterization of *CsPSY* and *CsUGT* promoters in *Crocus sativus* L.

**DOI:** 10.1371/journal.pone.0195348

**Published:** 2018-04-10

**Authors:** Archana Bhat, Sonal Mishra, Sanjana Kaul, Manoj K. Dhar

**Affiliations:** Genome Research Laboratory, School of Biotechnology, University of Jammu, Jammu, India; National Institute of Plant Genome Research, INDIA

## Abstract

The dried stigmas of *Crocus sativus* constitute the saffron, which is considered to be the costliest spice of the world. Saffron is valuable for its constituents, which are mainly apocarotenoids. In order to enhance the production of apocarotenoids, it is imperative to understand the regulation of apocarotenoid biosynthetic pathway. In *C*. *sativus*, although the pathway has been elucidated, the information regarding the regulation of the pathwaygenes is scanty. During the present investigation, the characterization of promoters regulating the expression of two important genes i.e. *CsPSY* and *CsUGT* was performed. We successfully cloned the promoters of both the genes, which were functionally characterized in *Crocus sativus* and *Nicotiana tabaccum*. *In silico* analysis of the promoters demonstrated the presence of several important *cis* regulatory elements responding tolight, hormonesand interaction with transcription factors (TFs). Further analysis suggested the regulation of *CsPSY* promoter by Abscisic acid (ABA) and that of *CsUGT* by Gibberellic acid (GA). In addition, we also observed ABA and GA mediated modulation in the expression of significant TFs and *CsPSY* and *CsUGT* transcripts. Overall, the study addresses issues related to regulation of key genes of apocarotenoid pathway in *C*.*sativus*.

## Introduction

*Crocus sativus*, commonly called saffron, belongs to the family Iridaceae. Saffron is one of the most fascinating cash crops due to its dried red stigma, which constitutes saffron of commerce [1]. Iran, Spain, India, Greece, Azerbaijan, Morocco and Italy dominate the world saffron production, with Iran and Spain producing 80% of the world crop. In India, saffron is mainly grown in the state of Jammu and Kashmir [2]. Saffron is a rich source of several therapeutically important carotenoids and apocarotenoids. Apocarotenoids play variety of roles in many biological processes, like flowering and fruit colour development [3], light harvesting and protection of photosynthetic machinery [4], act against biotic and abiotic stresses [5] and in biosynthesis of ABA and Strigolactone (SL) [6].

Apocarotenoids are non-polar isoprenoids that have alternate double and single bond pattern in their structure. Saffron contains mainly three types of chemical components, which include picrocrocin, safranal, and the bright yellow coloured carotenoids that are responsible for its bitter taste, aroma and color [7]. The carotenoid and apocarotenoid pathways share common biosynthetic steps commencing from isopentenyl phosphate (IPP) to zeaxanthin. The first rate-limiting enzyme of the pathway i.e. phytoene synthase (PSY), produces phytoene by condensation of two molecules of GGDP (Geranyl geranyl diphosphate). Following this, by several concomitant steps production of zeaxanthin (apocarotenoid precursor) occurs, which is finally converted to different apocarotenoids by oxidative cleavage [8]. In plant metabolism, glycosylation is an important tool for modification of the metabolites, more specificallysecondary metabolites. More often it has been observed that glucosylation is the last step of any biosynthetic pathway affecting the bioactivity, stability, subcellular localization and solubility [9]. In *Crocus* besides, Zeaxanthin diglucosides several other glycosides are collectively responsible for characteristic aroma [7]. In *C*. *sativus* two subsequent UGT mediated glycosylation steps are required for the conversion of insloluble crocetin and picrocrocetin cleavage products to soluble forms. Further studies on two reported *UGTs* (*UGTCs2* and *UGTCs3*) have thrown fresh light on stigma specific expression of *UGTCs2* [9].

Although, various genes for apocarotenoid biosynthesis have been identified in *Crocus* [9, 10, 11]. However, information about its regulation is scanty. Regulation of a gene at transcriptional level can be studied by analyzing activity of TFs, experimental conditions and by analyzing the upstream region i.e. promoter of a gene. Apart from the essential sites required for basal transcription, promoter contains several important *cis*-regulatory elements like TF binding sites, phytohormone response elements, light response elements, etc [12]. In order to understand the complex regulatory mechanisms of the pathway, identification of upstream interacting factors and functional characterization of the promoters of pathway genes is essential. In *Citrus*, Lu et al. [13] functionally characterized the *LYC-β* promoter and observed its light mediated regulation. Several transcription factors (RAP2.2, PIF and RIN) have also been reported to interact with the promoter of carotenoid/apocarotenoid pathway genes [14, 15,16]. The over-expression of *CsULT1* in *Crocus* calli induced the expression of *CsPSY*, *CsPDS*, *CsBCH*, *CsCCD4b* and CsCCD2 genes [17]. For the present investigation, the promoters of two important genes *CsPSY* and *CsUGT* of carotenoid/apocarotenoid biosynthetic pathway were selected. Characterization of *CsPSY* and *CsUGT* promoters will be helpful to better understand the regulatory mechanism of apocarotenoid biosynthesis in *Crocus*.

## Materials and methods

### Genomic DNA isolation and construction of genome walking libraries

The corms of *C*. *sativus* were collected from Pampore, Kashmir where the saffron is cultivated on a large scale. The plants were raised were raised from corms in the greenhouse of School of Biotechnology, University of Jammu during September to December 2016. The plant has been deposited in the Jammu University Herbarium under accession no. JUHB 15904. Genomic DNA was isolated from fresh leaves using CTAB method given by Doyle [18], with little modifications. The genomic DNA was further digested separately with *Eco*RV, *Dra*I, *Pvu*II and *Stu*I restriction enzymes. These enzymes resulted in blunt ended genomic DNA fragments, to which adaptor sequences were ligated, generating four different genome walker libraries. The constructed libraries were used to clone *CsPSY (CsPSYp)* and *CsUGT (CsUGTp)* promoters. The 5’ upstream regions of *CsPSY* and *CsUGT* genes were isolated by GenomeWalker^™^technique [19]. All the experiments were performed using manufacturer’s protocol provided with GenomeWalker Universal kit (Clontech laboratories).

### Isolation of CsPSY and CsUGT promoters

Genome walking PCR was performed using gene specific primers designed from 5’ end of *CsPSY* and *CsUGT* genes and adaptor primers AP1 and AP2, specific to ligated adaptor sequences (provided in GenomeWalker kit). All four genomic libraries were used as template for Genome Walking PCR. The primary PCR was set up using the GSP1 and AP1 and four different libraries as template. The primary PCR product was further diluted and used as template for secondary PCR, where AP2 and GSP2 were used as nested primer pair. The secondary PCR products were resolved on 1% agarose gel. All PCR programs were performed following manufacturers’ protocols. The primer sequences are listed in S1 Table.

### Cloning and analysis of promoter sequence

All amplified products obtained from secondary PCR were purified from agarose gel using a QIAquick Spin kit (Qiagen) and further cloned into the pTZ57R/T cloning vector system using TA cloning kit (Fermentas). Plasmids were isolated from different clones and were then processed for sequencing after purification. For removal of vector contamination, sequences were trimmed using VecScreen software (https://www.ncbi.nlm.nih.gov/tools/vecscreen/) and were aligned by BLASTn and ClustalW program. The presence of putative *cis*-regulatory elements were detected by PLACE (http://www.dna.affrc.go.jp/PLACE/signalscan.html) and Plant CARE (http://bioinformatics.psb.ugent.be/webtools/plantcare/html) scan tools [13]).

### Agrobacterium mediated transient assay in heterologous and homologous system

*Agrobacterium tumefaciens* strain GV3101 transformed with pCAMBIA1391Z, pCAMBIA1391Z-*CsPSYp*::*GUS* and pPZP200N-*35S*::*GUS* were inoculated in 5 ml of LB medium containing Kanamycin (50 μg/ml), Rifampicin (50 μg/ml) and Gentamycin (40 μg/ml). The cultures were incubated at 28°C for two days at 120 rpm. Similarly, pMDC164, pMDC164-*CsUGTp*::*GUS* and pPZP200N-*35S*:: *GUS* transformed *A*. *tumefaciens* strain GV3101 were inoculated in the above-mentioned medium. 10 μl of primary culture was inoculated in 50ml of LB medium containing 10 mM MES, 30 μM acetosyringone and above-mentioned antibiotics. The secondary culture (OD 0.6) was syringe-infiltrated on abaxial side of leaves of *Nicotiana tabaccum*. The infiltrated leaves were incubated for two days and further processed for estimation of GUS activity. Similarly, *Crocus* calli were co-cultivated with *A*. *tumefaciens* GV3101 harbouring the above-mentioned constructs and were kept on MS media. After two days of incubation, calli were transferred to selective MS media containing Cefotaxime (250 μg/ml), Kanamycin (100 μg/ml), TDZ (1mg/l) and IBA (0.5 mg/l). Plates were incubated at 15°C for one monthand further processed for GUS activity.

### Histochemical GUS staining

Post-infiltrated tobacco leaves and co-cultivated *Crocus* calli were processed for GUS activity, following manufacturer’s protocol (GUSS-1KT, 1002229482-Sigma). Briefly, detached leaves were kept in fixative buffer solution and incubated for 45 min at 25°C followed by washing. Samples were vacuum infiltrated for 2–5 minutes in staining solution and incubated overnight at 37°C. Subsequently, samples were washed with 75% ethanol. Similarly *Crocus* calli were processed for GUS activity detection.

### Abscisic acid and Gibberellic acid treatment

Two months old *Crocus* plants and calli were treated with ABA (100 μM) and GA (100 μM). For ABA treatment 100 μM aqueous solution was prepared and poured in the pots in which saffron corms were growing. For GA treatment 100 μM solution was prepared in water to which 10% Tween 20 was added. GA solution was sprayed over the plants. These plants were covered with perforated autoclave bags. For mock treatments, plants were treated with autoclaved water (in case of ABA) and water containing 10% Tween 20 (in case of GA). Flowers of treated plants were harvested after 12 and 24 hours along with mock treated plants. The seven days post-treated calli were also harvested along with mock and processed further.

### RNA isolation and real time PCR

Total RNA was extracted from flower and calli of *Crocus* using Trizol reagent (Invitrogen). The cDNA was synthesized from each RNA sample following manufacturer’s protocol (GoScript Reverse Transcription System-A5000). The estimation of qRT-PCR based quantitative fold induction was performed using the SYBR Green (Power Syber Green 4367659) and ABI StepOne Real time (Applied Biosystems). Experiments were conducted in three biological and three technical replicates. The reaction was carried out in a total volume of 10 μl that comprised of 5 μl of 2X SYBR Green Master Mix, gene specific primer pairs (0.2 μM each) and 20 ng of cDNA template. The cycling parameters were 95°C for 20 s, followed by 40 cycles of 95°C for 15 s and 60°C for 1 min. The ΔΔ^-CT^ method was used for relative quantification and ACTIN2 (ACT2) was used as an internal control. Primers for RT-PCR were designed from unique region of the selected transcripts as given in S1 Table.

## Results

### Isolation of *CsPSY* and *CsUGT* promoters

PCR based Genome walking was performed, using all the four genomic libraries which resulted in products of different lengths for *CsPSYp* and *CsUGTp*. The largest amplicons of *CsPSYp* (900bp) and *CsUGTp* (850 bp) were obtained from *Eco*RV and *Pvu*II genomic library, respectively (S1 and S2 Figs). The isolated sequences showed alignment at 5’ region of *CsPSY* (AY262037.1) and *CsUGT* (AJ888514.1) sequences, respectively.

### In silico analysis of *CsPSY* and *CsUGT* promoters

**Fig 1:**
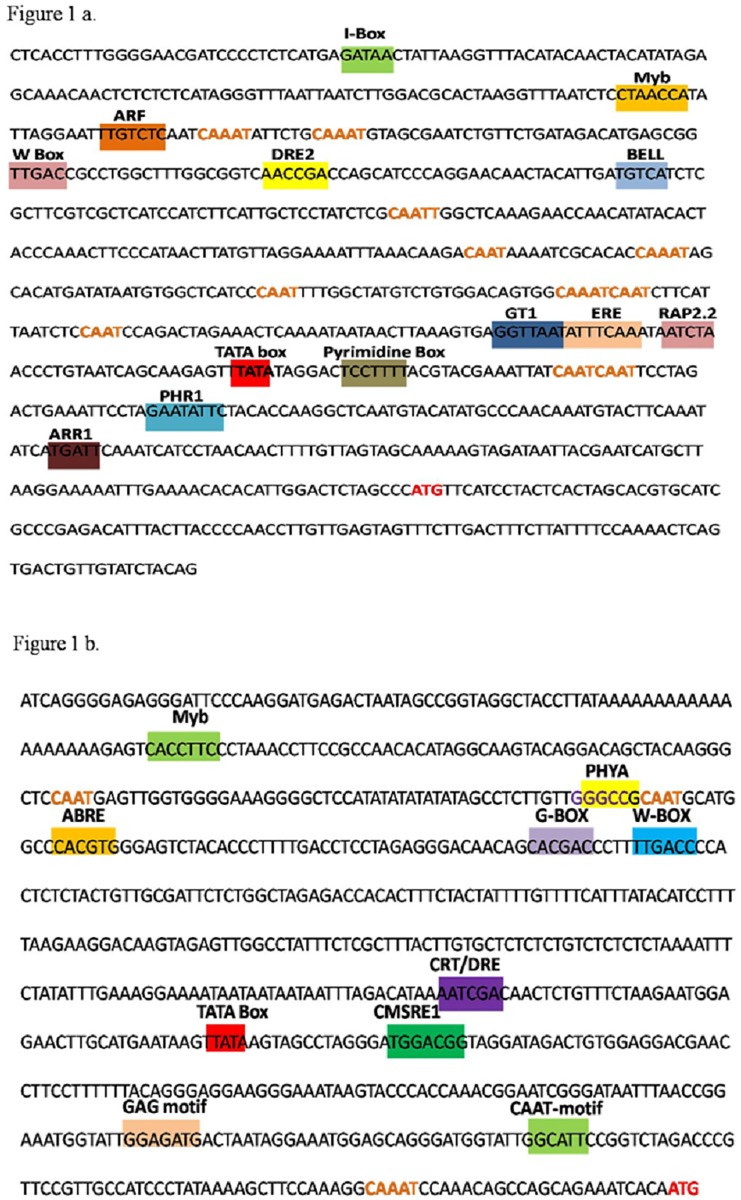
**A. The 5’ upstream promoter sequences of the *CsPSY* gene**. Some important *cis*-regulatory elements present in *CsPSYp* are highlighted in different color. Putative TATA box and transcription start sites are shown in red color. **1B. The 5’ upstream promoter sequences of the *CsUGT* gene**. Some important *cis*-regulatory elements present in *CsUGTp* are highlighted in different color. Putative TATA box and transcription start sites are shown in red color.

*In silico* analysis showed presence of various important *cis*-regulatory elements involved in different signaling cascades. The analysis of the promoter sequences demonstrated presence of light responsive, TF binding and hormone signaling elements. Besides these, other elements involved in signaling network for phosphate starvation, carbohydrate metabolism and defense response were also revealed by this analysis (Fig 1a and 1b). In case of *CsPSYp*, prediction analysis exhibited the presence of a potential TATA box 213 bp upstream to ATG and 27 bp upstream to TSS (+1). In *CsUGTp*, a potential TATA box, observed at 241 bp upstream to ATG and 27 bp upstream to TSS (+1) was predicted. A detailed description about *cis* regulatory elements present in *CsPSYp* and *CsUGTp* are given in Tables 1 and 2.

**Table 1:** Predicted *cis*-regulatory elements in *CsPSY* promoter.

Element	Element Core Sequence	Element Number	Function
GATABOX	GATA	4	Light responsive element
CAREOSREP1	CAACTC	1	Cystein proteinase regulatory element
CACTFTPPCA1	YACT	1	Mesophyll expression module regulatory element
MYBATRD22	CTAACCA	1	MYB TF binding site
CPBCSPOR	TATTAG	1	Cytokinin enhanced protein binding element
SEBFCONSSTPR10A	YTGTCWC	1	Involved in pathogenesis
ARFAT	TGTCTC	1	Auxin response factor
CAATBOX1	CAAT	1	CAAT promoter consensus sequence
WBOXATNPR1	TTGAC	1	WRKY TF binding protein
DRE2COREZMRAB17	ACCGAC	1	DBF1 and DBF2 binding element
CBFHV	RYCGAC	1	Dehydration-responsive element binding proteins
BIHD1OS	TGTCA	1	BELL homeodomain binding site
GT1CONSENSUS	GRWAAW	1	Light responsive element
CARGATCONSENSUS	CCWWWWWWGG	1	FLC binding site
ABRERATCAL	MACGYGB	1	ABRE-related sequence
TATAPVTRNALEU	TTTATATA	1	TATA-like motif
PYRIMIDINEBOXOSRAMY1A	CCTTTT	1	Involved in sugar repression
P1BS	GNATATNC	1	Phosphate starvation response 1 element
ARR1AT	NGATT	1	ARR1-binding element
Box 4	TTATTT	1	Part of a conserved DNA module involved in light responsiveness
Box III	CATTTACACT	1	Protein binding site
CAAT-box	CAAAT	20	Common *cis*-acting element in promoter and enhancer regions
ERE	ATTTCAAA	1	Ethylene responsive element
ATCTA motif	ATCTA	2	RAP2.2 binding site
GT1-motif	GGTTAAT	1	Light responsive element
MBS	CGGTCA	1	MYB Binding Sit
Skn-1_motif	GTCAT	1	Involved in endosperm expression
TATA-box	TATA	7	Core promoter element around -30 of transcription start
W box	TTGACC	1	WRKY TF binding site

**Table 2:** Predicted *cis*-regulatory elements in *CsUGT* promoter.

Element	Element Core Sequence	Element Numbers	Function
ARR1AT	NGATT	1	ARR1 response regulator
SURECOREATSULTR11	GAGAC	1	Sulfate uptake; sulfate transporter; ARF; -S; S
DOFCOREZM	AAAG	4	Core site required for binding of Dof proteins
BOXLCOREDCPAL	ACCWWCC	1	MYB; R2R3 type; PAL: Elicitor; UV-B; Dilution
CANBNNAPA	CNAACAC	1	Involved in seed storage protein
SORLIP2AT	GGGCC	1	PhyA response element
ABRE	CACGTG	1	Involved in the abscisic acid responsiveness
Box-W1	TTGACC	1	Fungal elicitor responsive element
CAAT-box	CAAT	6	Common *cis*-acting element in promoter and enhancer regions
CBFHV	RYCGAC	1	CBF/AP2 domain binding site
CMSRE1IBSPOA	TGGACGG	1	Carbohydrate Metabolite Signal Responsive Element 1
CATT-motif	GCATTC	1	Light responsive element
G-Box	CACGTG	1	Light responsive element
G-box	CACGAC	1	Light responsive element
GAG-motif	GGAGATG	1	Light responsive element
GTGGC-motif	GATTCTGTGGC	1	Light responsive element
P-box	CCTTTTG	1	Gibberellin-responsive element
TATA-box	ATATAT	21	Core promoter element around -30 of transcription start
W box	TTGACC	2	WRKY TF binding site

### *CsPSY* and *CsUGT* promoter activity assay

**Fig 2:**
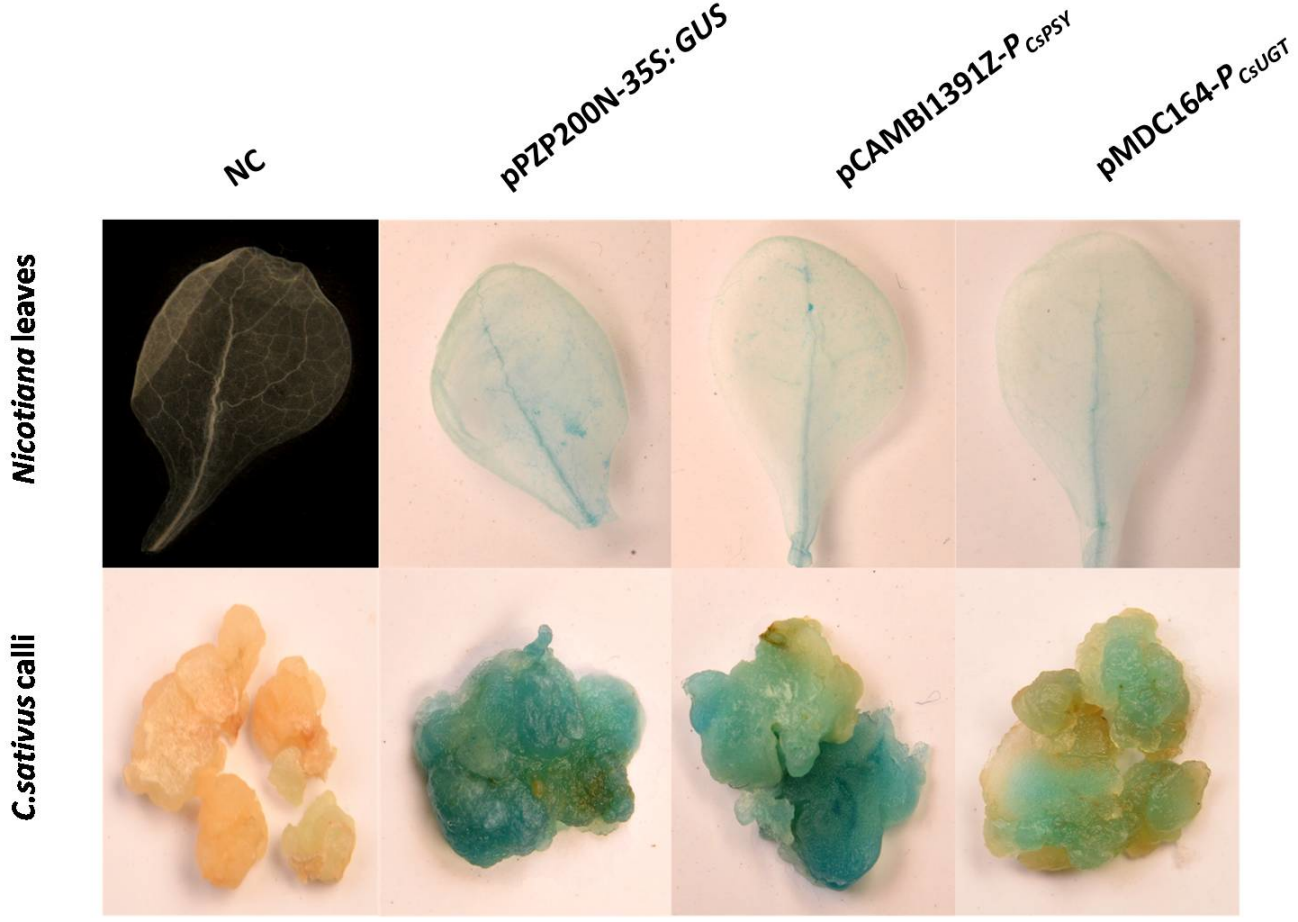
Histochemical analysis of GUS activity. **(Up)**—For qualitative analysis, infiltrated tobacco leaves (with respective construct) and **(Down)** transformed *Crocus* calli (with respective construct) were separately subjected to histochemical GUS staining.

GUS activity was detected in pCAMBIA1391Z-*CsPSYp*::*GUS* and pMDC164-*CsUGT*::*GUS* (Promoter-Reporter construct) infiltrated leaves while in mock treated plants no GUS activity was observed. The transformed *Crocus* calli harbouring pCAMBIA1391Z-*CsPSYp*::*GUS* and pMDC164-*CsUGTp*::*GUS* showed active GUS expression as compared to negative control (pCAMBIA1391Z/pMDC164) (Fig 2).

### Effect of ABA and GA on *CsPSY* and *CsUGT* promoter activity

**Fig 3:**
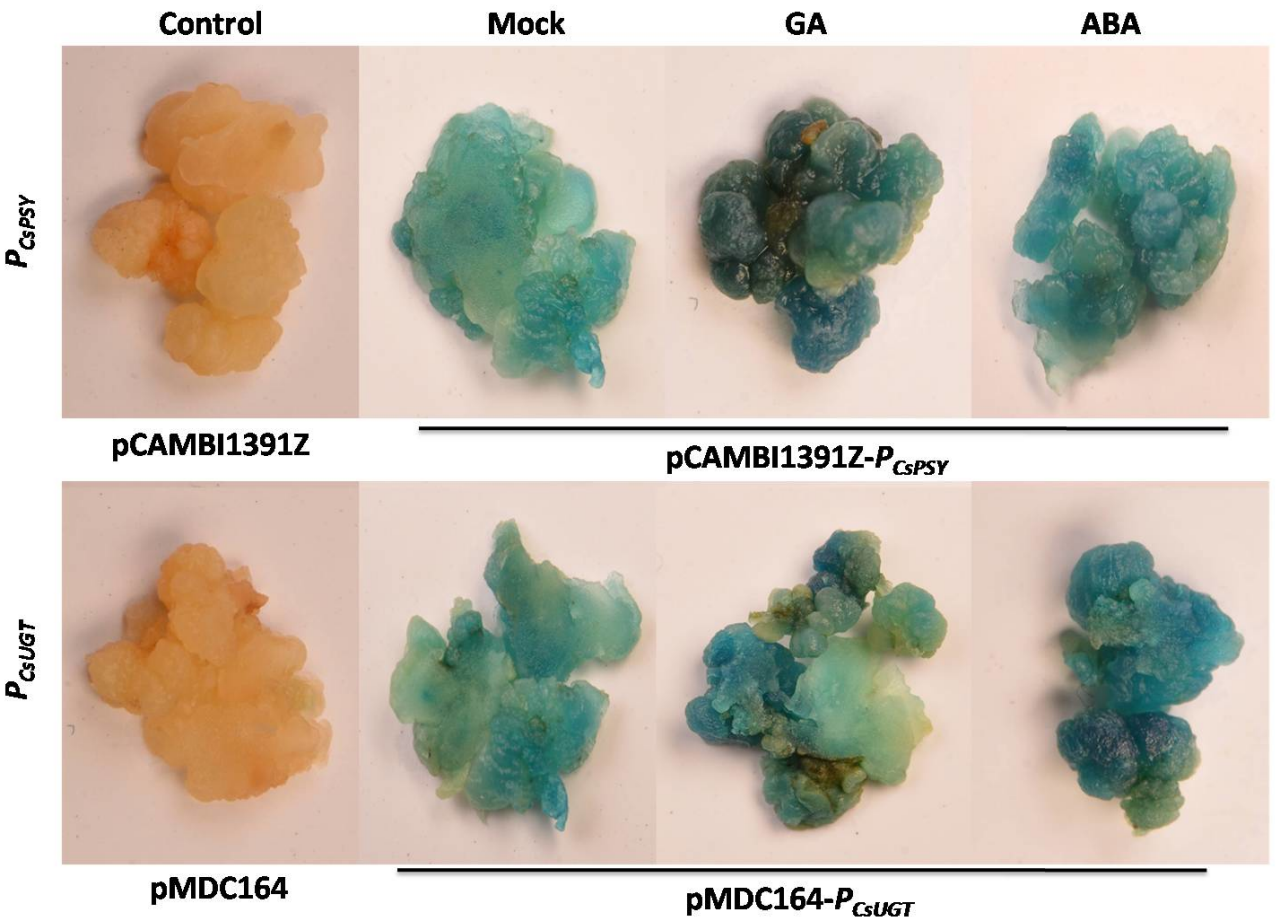
Effect of ABA and GA on *CsPSY* and *CsUGT* promoter activity. For qualitative analysis, ABA and GA treated **(Up) *CsPSYp* (Down) *CsUGTp*** transformed *Crocus* calli were separately subjected to histochemical GUS staining.

*In silico* analysis as reported above demonstrated the presence of ABA and GA responsive elements (ABRE and P-Box) in both *CsPSYp* and *CsUGTp*. Several reports suggest that GA and ABA treatment modulates the expression of a number of transcripts either involved in biosynthesis or other signaling cascades. Recently, Mandal et al. [20] have shown positive regulation of ARM protein by GA treatment. In the present case, the ABA and GA treatment enhanced *CsPSYp* and *CsUGTp* driven GUS activity as compared to mock treated calli (Fig 3). However, GUS activity was comparatively high in case of *CsPSYp* transformed calli compared to *CsUGTp* transformed calli after ABA and GA treatment. It was also observed that in *CsUGTp* transformed calli GUS activity was higher in case of ABA as compared to GA treatment.

### Effect of ABA and GA on expression of *CsPSY* and *CsUGT* genes

**Fig 4:**
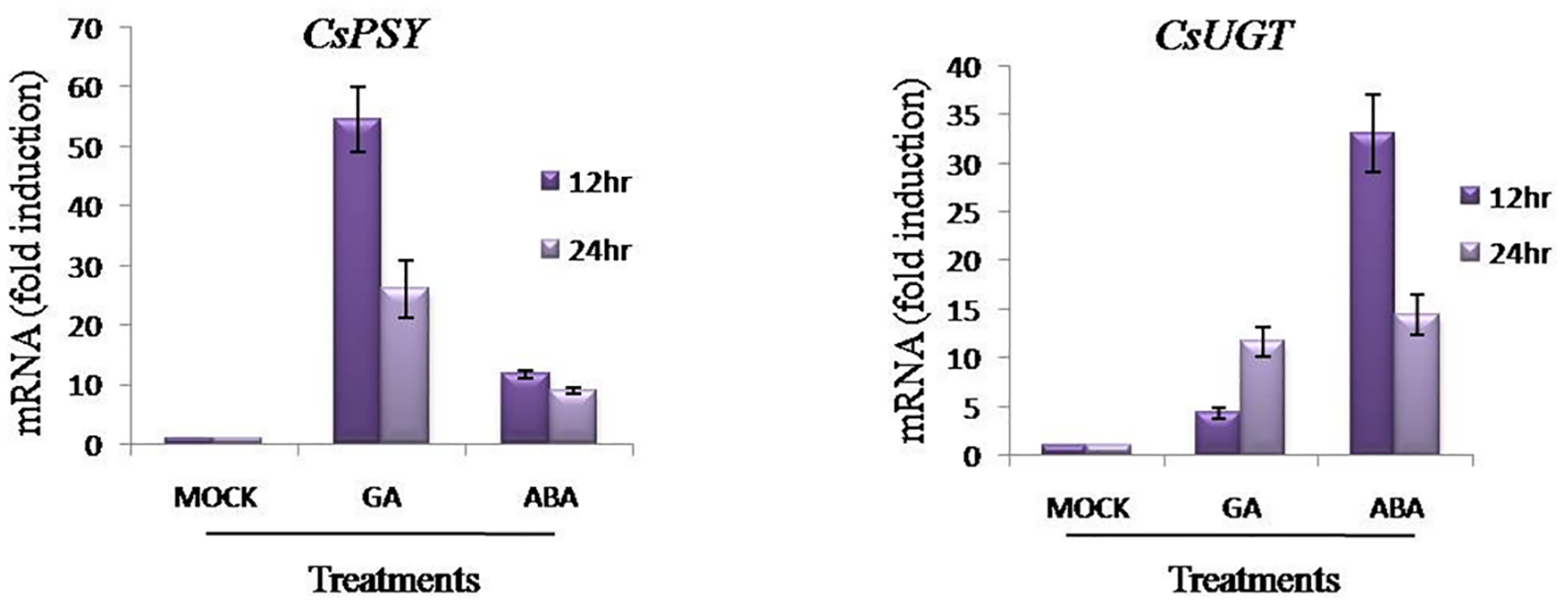
Relative expression of *CsPSY* and *CsUGT* after ABA and GA treatment. qRT-PCR analysis of ***CsPSY*** and ***CsUGT*** transcripts in ABA and GA treated flowers and leaves of *C*.*sativus* at 12 and 24 hrs post treatment.

Relative expression analysis of *CsPSY* and *CsUGT* was performed in flower and leaves of *C*. *sativus* after ABA and GA treatment. As shown in Fig 4, a significant up-regulation of *CsPSY* and *CsUGT* transcripts was observed after ABA and GA treatment as compared to mock. Interestingly, the peak expression was observed in flowers 12 hrs post-GA treatment. However, the *CsUGT* was highly up-regulated in flowers 12 hrs post-ABA treatment.

### Effect of ABA and GA on expression of transcription factors

**Fig 5:**
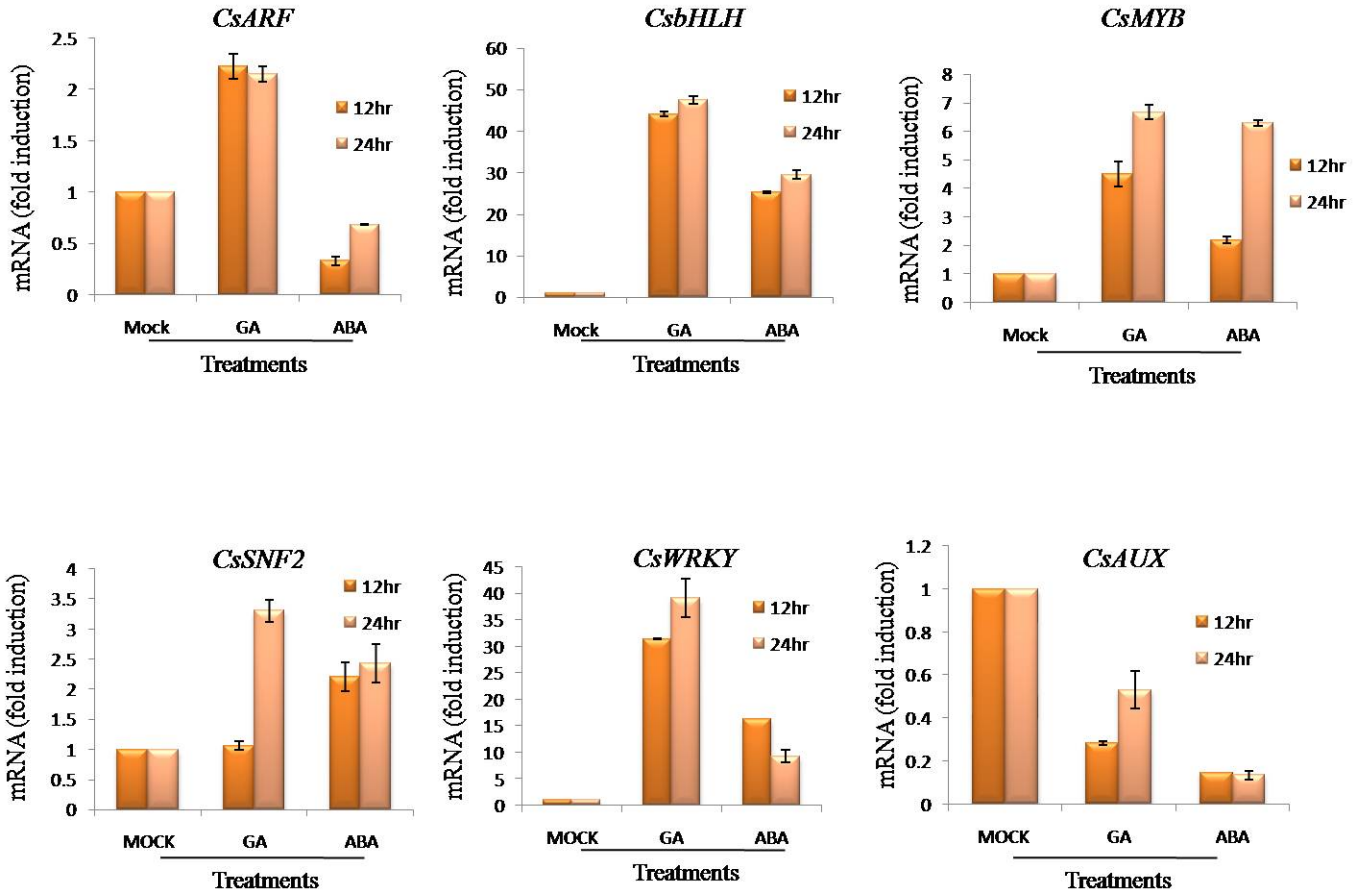
Relative expression of *TFs* preferably expressing in stigma. qRT-PCR analysis of *CsbHLH*, *CsWRKY*, *CsAUX*, *CsSNF*, *CsARF* and *CsMYB* transcripts in ABA and GA treated flowers of *C*.*sativus* at 12 and 24 hrs post treatment.

Six transcription factors i.e. *CsbHLH*, *CsARF*, *CsMYB*, *CsWRKY*, *CsSNF* and *CsAUX* were selected for expression analysis after 12 and 24 hrs in ABA and GA treated flower samples. Significant modulation in expression of these TFs was observed. The *CsbHLH* exhibited remarkable modulation after ABA and GA treatments in flowers. The up-regulation was consistent at both time points and was comparatively higher in GA treated flowers. The *ARF* demonstrated high transcript accumulation in GA treated flowers at 12 and 24 hours post treatment. *MYB* showed increased expression after GA and ABA treatments as compared to mock. However, expression was high in GA treated flowers as compared to ABA. Relative expression of *SNF* followed almost similar pattern as for *MYB*. While *WRKY* showed aninteresting pattern of expression; its expression increased in 24 hrs post-GA treatment whereas in ABA treated flowers expression decreased in 24 hrs post treatment. In contrast, the expression of *AUX* was downregulated after GA and ABA treatments (Fig 5).

## Discussion

The understanding of regulation of various genes and enzymes involved in carotenoid/apocarotenoid pathway may help in its engineering to enhance the production of various bioactive compounds. One of the possible means in *C*.*sativus* involves the overexpression and functional knock-out of apocarotenoid pathway genes [21]. Other interesting way is to explore the regulatory regions of native promoters and utilize the generated information to attain desired metabolite production. In *C*. *sativus*, characterization of promoters of *CsCCD2* and *CsLycB2a* has been reported earlier [10]. The *PSY* and *UGT* are the important enzymes responsible for apocarotenoid production. In some plants such as tomato, citrus and sweet pepper, light and development dependent expression of *PSY* and *UGT* genes and its correlation with apocarotenoid content has been demonstrated [12, 1]. In citrus fruit, *PSY* transcript level increases with rise in fruit carotenoid content [22]. Two *UGT* isoforms (*UGTCs2* and *UGTCs3*) in *C*. *sativus* have been identified [9]. Among them, higher expression of *UGTCs2* in stigma was observed, which was further correlated to crocetin production [9]. Therefore, for the present investigation we explored the promoter regions of *PSY* and *UGT* genes genes so as to identify characteristic elements. Detailed information about various important genes of carotenoid and apocarotenoid biosynthetic pathway and related transcription factors in *C*. *sativus* was extensively reviewed recently [1].

### Cis-regulatory elements in *CsPSY* and *CsUGT* promoters

**Fig 6:**
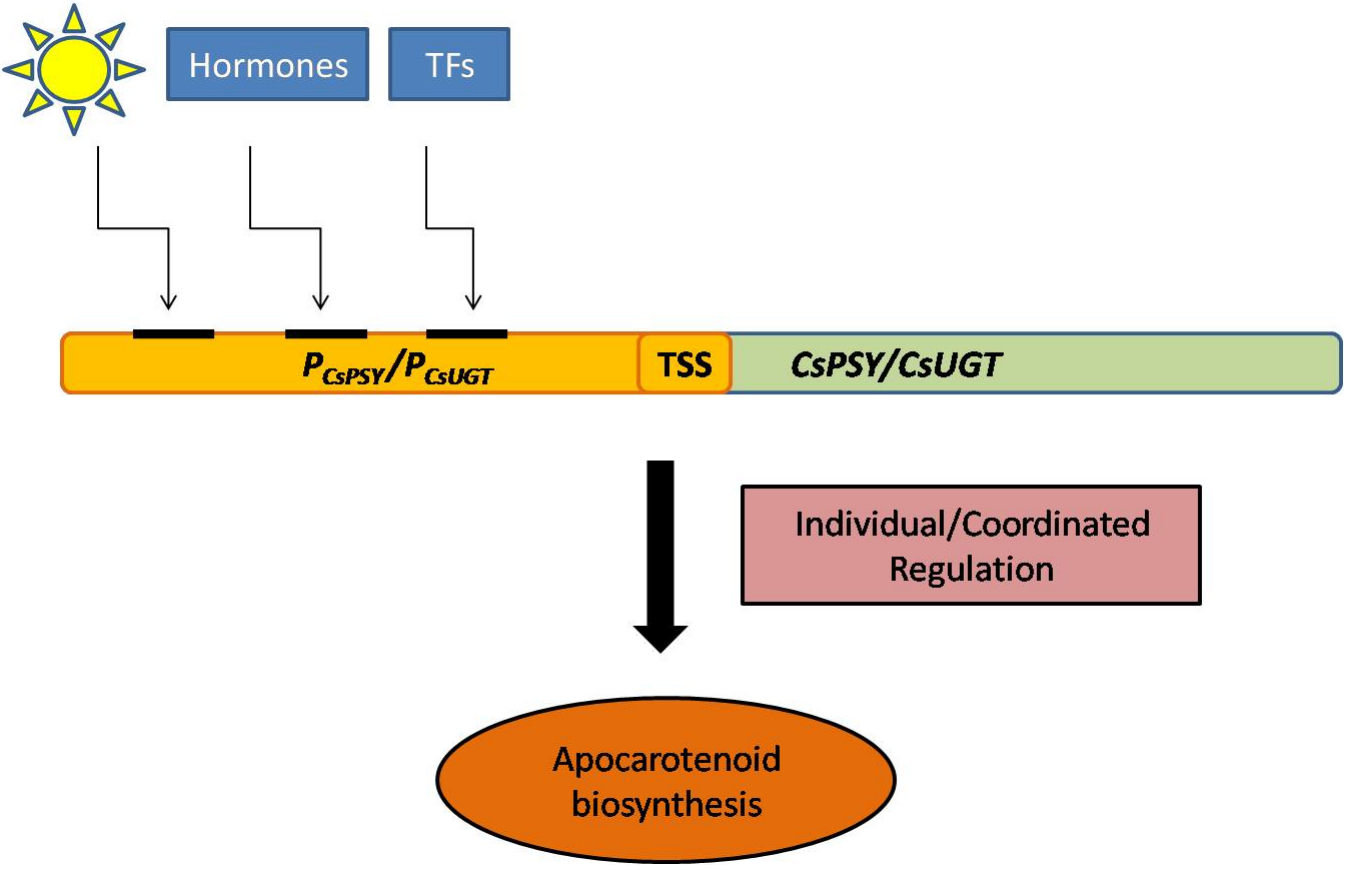
Probable mechanism of regulation. Picture showing probable regulatory mechanism of *CsPSY* and *CsUGT* promoter activity.

*In silico* analysis of *CsPSY* and *CsUGT* promoters showed the presence of diverse *cis*-regulatory elements involved in different signaling cascades. The active form of promoter was validated by the presence of TATA box and transcription initiation site. W-Box element which is a potential binding site of WRKY group of TFs, was present in promoters of *CsPSY* and *CsUGT* genes. According to Mandal et al. [23] WRKY group of TFs regulates the genes involved in plant development, by binding to W-Box elements present in their promotersunder stress condition. The light responsive elements present in *CsPSY* and *CsUGT* promoters have been predicted to be involved in different light regulated signaling response in many plant species [15]. In *CsPSY* promoter presence of light responsive elements viz. GATA box, CAAT box, GT1 and Box 4 was observed. GATA box is a GATA-binding site and is involved in light-regulated developmental cues [24]. GT1 is important *cis*-regulatory element involved in control of light regulated transcription [12]. In *CsUGT* promoter, light responsive *cis*-elements like SORLIP2AT (Phy A response element), CATT motif, G-boxand GAG motif were present. SORLIP2AT element has been reported to be involved in phytochromeA regulated gene expression [25]. The G-box element is responsible for PIF (bHLH) binding, a central mediator in a variety of light-mediated responses and also in repression of photomorphogenic development in dark [15]. Saffron is a geophyte with a very short aboveground growth period. It breaks its dormancy in winter resulting in shoot and flower bud formation. Stigma development starts in unopened flower bud. Initially the stigma is yellowish, followed by orange and acquires scarlet colour during the last stage i.e. fully bloomed flower. At scarlet stage the amount of apocarotenoids is the highest. Presence of light responsive *cis*-elements in the *CsPSYp* and *CsUGTp* provide a clue about the regulation of the genes involved in carotenoid/apocarotenoid pathway. Thus, it can be predicted that for the formation of scarlet stigma, besides, TFs and phytohormones, light is a necessary factor, which triggers the expression of pathwaygenes, resulting in the maximum concentration of apocarotenoids (Fig 6).

Besides, several elements required for phytohormone mediated signaling were also present in *CsPSYp* (ABRE, ERE and GARE) and *CsUGTp* (ARR1, ABRE and P-Box). The ABRE element has earlier been identified in the *PSY* promoter of *Oryza sativa* and *Zea mays* [26, 27, 28]. Besides, ABRE has been reported to play a crucial role in the regulation of NAC TFs under stressed condition [29]. The phytohormones play significant role in development and defense and hence presence of these elements in *CsPSYp* and *CsUGTp* point towards the possibility of their phytohormone mediated regulation. In *C*. *sativus*, flower development and maturation is correlated with its metabolite production, therefore it is likely that ABA, GA, ethylene and auxin treatment may also modulate metabolite levels by influencing the expression of *CsPSY* and *CsUGT* transcripts. In current study, the induced expression of *CsPSY* and *CsUGT* was also observed after ABA and GA treatment in *C*. *sativus* flowers. The probable reason for this induced expression is the presence of ABA and GA phytohormone binding factors in the promoter sequence of *CsPSY* and *CsUGT* genes. Presence of these binding factors may have resulted in increased transcription of these genes.

Transcription factors are other imperative candidates involved in biosynthetic pathway regulation, which affect the expression of pathway genes under different conditions. *CsPSYp* and *CsUGTp* sequences showed binding sites for MYB, MYC, MADS, CBF, WRKY, BELL homeodomain, ARF, RAP2.2, transcription factors. Besides, *CsPSYp* and *CsUGTp* contain sites involved in carbohydrate metabolic signaling (Pyrimidine box, CMSRE1IBSPOA), fungal elicitor responsive element (Box-W1) andphosphate starvation response etc. As *CsUGT* is involved in glucosylation, the presence of carbohydrate metabolic signaling element in *CsUGTp* seems reasonable. Earlier workers have suggested that sugar accumulation in *Crocus* corms is positively correlated with flower development [30]. Hence, it can be argued that presence of sugar responsive element might be a part of carbohydrate metabolic signaling in relation to flower development. In cotton, promoter of *GhGlcAT1* gene was responsive to glucose and sucrose as their treatment increased the expression of GUS fusion protein as compared to other sugars [25]. Therefore, by analyzing these elements one can identify the upstream candidates involved in regulation of gene expression like hormones, TFs, etc. under such conditions.

### ABA and GA regulates *CsPSY* and *CsUGT* promoter activity

The presence of ABA and GA responsive elements in *CsPSY* and *CsUGT* suggested the ABA and GA mediated regulation of gene expression. In accordance, the enhanced GUS expression in *Crocus* calli and tobacco leaves was observed after hormone treatments. These results thus justify the functional relevance of ABA and GA responsive elements in *CsPSYp* and *CsUGTp*. Again, the enhanced expression of *CsPSY* and *CsUGT* transcripts in ABA and GA treated flowers and leaves further validated the impact of these hormones on the activity of *CsPSYp* and *CsUGTp*. Expression of *PSY* is highly regulated by stress, development and light signaling [31, 32]. Apart from this, as *PSY* catalyzes the rate, limiting step of this pathway so its modulation can affect carotenoid metabolic flux. Dong et al. [33] reported increase in the transcript level of two closely related homologs of *UGT* (*UGT71B7* and *UGT71B8*) in ABA treated *Arabidopsis*. Thus, understanding of the regulatory aspects of PSY promoter may help to manipulate the carotenoid/apocarotenoid biosynthesis. In present study we reported that initially at 12 hours of post GA and ABA treatments *CsPSY* expression was induced. Further *CsPSY* transcript level decreased after 24 hrs of post GA and ABA treatment. Probably, by regulatory feedback inhibition is responsible for down regulation of *CsPSY* transcript as earlier mentioned by Zeng et al. [12].

### ABA and GA modulates the expression of TFs

The possible binding of various transcription factors to the promoter of the pathway genes has been reported to modulate their expression. In this study, the analysis of *CsPSYp* and *CsUGTp* revealed presence of binding elements for many regulatory agents including transcription factors. Moreover, there are other reports demonstrating that TFs may itself be regulated by phytohormones. To work on this possibility, six potential transcription factors preferentially expressed in stigma [34] and having potential binding sites in *CsPSY* and *CsUGT* promoters were selected for expression analysis. The significant modulation in expression of these TFs in *C*. *sativus* suggests co-regulation of transcription factors and apocarotenoid biosynthestic pathway genes. As shown in Fig 5 the relative expression pattern of *CsARF*, *CsbHLH*, *CsMYB*, *CsSNF* and *CsWRKY* transcription factors was almost similar as in case of *CsPSY* and *CsUGT* transcripts after GA and ABA treatment. ARF and Aux TFs are the components of Auxin signaling pathway where Aux TF represses activity of ARF TF. It has been reported in literature that Auxin treatment decreases the level of Aux TF. According to Bjorklund S and Wang T, GA and ABA treatment increases Auxin biosynthesis and polar auxin transport [35, 36]. So this justifies that GA and ABA treatment decreases the level of Aux TF by enhancing the Auxin biosynthesis. Again a correlation between expression of *CsbHLH* and *CsPSY* transcript can be observed as the expression of *CsPSY* decreased at 24 hours post GA and ABA treatments (Fig 4) while *CsbHLH* TF showed increased expression in GA and ABA treated flowers (Fig 4) at similar time point (24 hours). Toledo-Ortiz et al. [14] reported suppression of PSY transcript by PIF a *CsbHLH* transcription factor in *Arabidopsis thaliana* which justifies above mentioned correlation between *CsbHLH* and *CsPSY* transcripts. Earlier, Gómez-Gómez et al. [37] identified a MYB TF in *C*. *sativus* whichhas been demonstrated to play role in the regulation of stigma morphology. The wound inducible *PsWRKY* of *Papaver somniferum* has been reported to enhance benzylisoquinoline alkaloid biosynthesis metabolism, which also exhibited modulation after MJ and ABA treatment [38]. This TF is known to play significant role in hormone signaling pathways and metabolism [39]. Further, most of the TFs utilized in this study are known to regulate secondary metabolism [40] and are specific to stigma [33]. Hence, possibly these TFs may be regulating apocarotenoid biosynthesis in *Crocus* stigma.

## Conclusion

The present study elucidates the functional validation of *CsPSY* and *CsUGT* promoter sequences in heterologous as well as homologous systems, thereby enhancing our understanding of the regulation of apocarotenoid biosynthetic pathway. Based on the results of various experiments, it appears that ABA and GA may be regulating the expression of these promoters. Besides, increased expression of stigma specific TFs was also observed in response to ABA and GA. Co-regulation of TFs, *CsPSY* and *CsUGT* promoters by ABA and GA is a pointer towards their important role in regulation of apocarotenoid pathway genes in *C*.*sativus*. So, for further studies to understand regulatory signaling mechanism of Apocarotenoid biosynthetic pathway bHLH, WRKY and MYB TFs can be potential candidates. This study can prove to be highly useful to identify upstream *trans*-acting elements of these promoters as well as for genetic engineering to enhance metabolite production in saffron.

## Supporting information

S1 Fig Construction of genomic DNA library. *Dra*I, *Eco*RV, *Pvu*II and *Stu*I digested and purified libraries.
(JPG)

S2 Fig Isolation of *CsPSY* and *CSUGT* promoters. Amplification of *CsPSYp* and *CsUGTp* with specific primers using *Dra*I, *Eco*RV, *Pvu*II and *Stu*I digested libraries as templates.
(JPG)

S1 Table List of the primers used for study. (DOCX)
