# Supplementary figures and images for: Elucidation and functional characterization of CsPSY and CsUGT promoters in Crocus sativus L
Source: PLoS One. 2018 Apr 10;13(4):e0195348. doi: 10.1371/journal.pone.0195348 (PMC5892871; doi:10.1371/journal.pone.0195348)

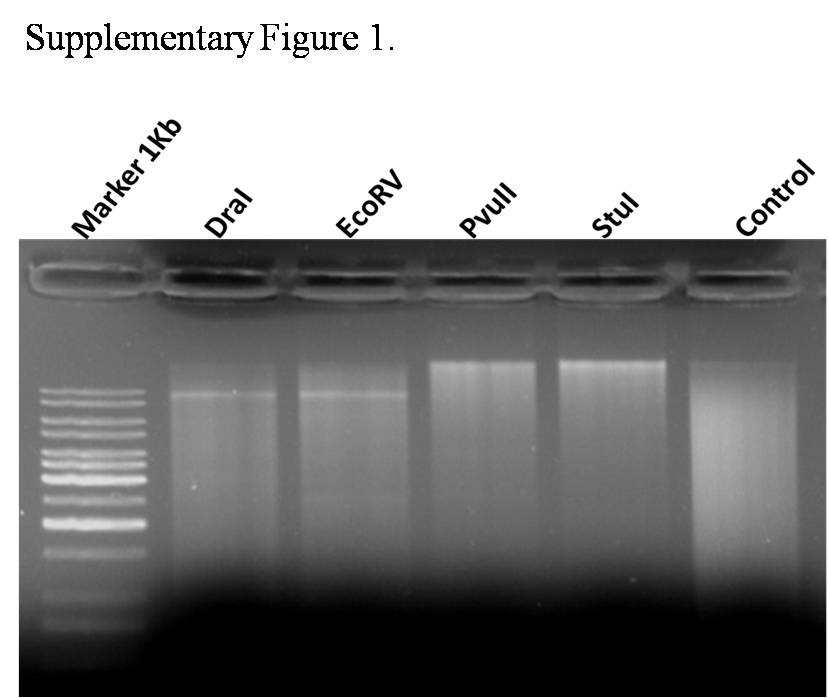

Supplement: S1 Fig — DraI, EcoRV, PvuII and StuI digested and purified libraries. (JPG) [file pone.0195348.s001.jpg]

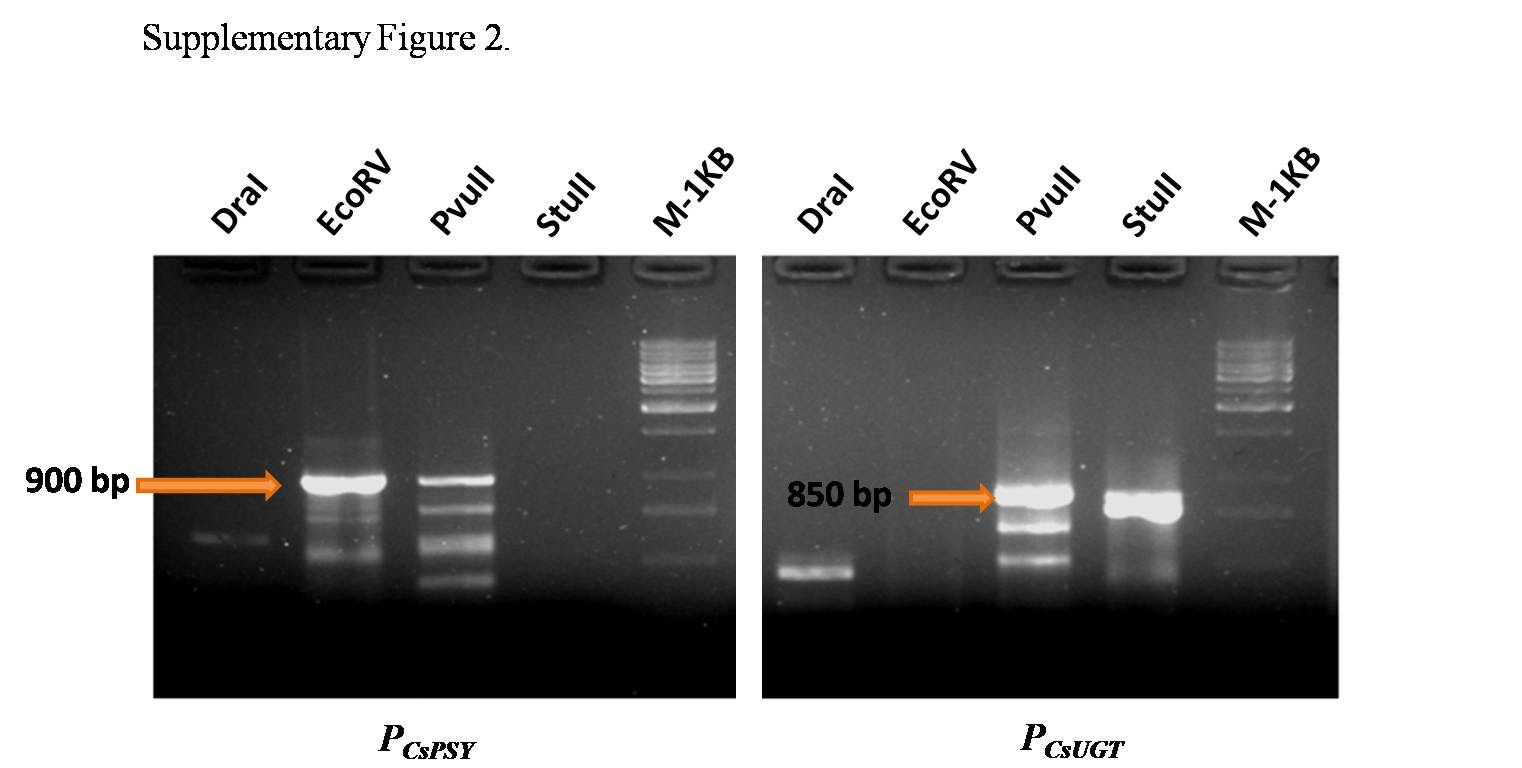

Supplement: S2 Fig — Amplification of CsPSYp and CsUGTp with specific primers using DraI, EcoRV, PvuII and StuI digested libraries as templates. (JPG) [file pone.0195348.s002.jpg]
