# Supplementary material for: Elucidation and functional characterization of CsPSY and CsUGT promoters in Crocus sativus L
Source: PLoS One. 2018 Apr 10;13(4):e0195348. doi: 10.1371/journal.pone.0195348 (PMC5892871; doi:10.1371/journal.pone.0195348)
Supplement: S1 Table — (DOCX) [file pone.0195348.s003.docx]

| Primer Code | Sequence | Purpose |
| --- | --- | --- |
| *CsUGTP1* | 5’ TGTTGACGAGTGTGGTGAGGAGGTTG 3’ | Promoter isolation using GenomeWalking |
| *CsUGTP2* | 5’ GTTGATATGGCCTTGTGCTGGACAGG 3’ |  |
| *CsPSYP1* | 5’ GGTATCAGACAAGGCAGCATCGAACATG 3’ |  |
| *CsPSPY2* | 5’ AAGATGCATTGGGTCCATCTACAAGCTC 3’ |  |
| *WA1* | 5’ GTAATACGACTCACTATAGGGC 3’ |  |
| *WA2* | 5’ ACTATAGGGCACGCGTGGT 3’ |  |
| *CsUGTpMDC164F* | 5’ CACCGATTCCCAAGGATGAGACTA 3’ | Gateway Cloning of CsUGT promoter |
| *CsUGTpMDC164R* | 5’ GCCTTTGGAAGCTTTTATAG 3’ |  |
| *CsPSYpcamF* | 5’ GGATCCGATCCCCTCTCATGAGATA 3’ | pCAMBIA Cloning of *CsPSY* promoter |
| *CsPSYpcamR* | 5’ GTCGACCAACAGTCACTGAGTTTTGG 3’ |  |
| *CsUGTF* | 5’ ACCAGCCTATGAATGCCAAGTAT 3’ | RT expression of *CsUGT* gene |
| *CsUGTR* | 5’ ACCTTGCGGCATTCTCTCTAAT 3’ |  |
| *CsPSYF* | 5’ TTCGATGCTGCCTTGTCTGA 3’ | RT expression of *CsPSY* gene |
| *CsPSYR* | 5’ CCTTGAAGGGCTGAATGTCAA 3’ |  |
| *CsARF-F*  *CsARF-R (CsTc012551)* | 5’ CTCCGAAGAGCTCTTTCATG 3’  5’ CAATCAAGAGAGCAGCAAAC 3’ | RT expression of Transcription factors of *Crocus sativus* |
| *CsAux-F*  *CsAux-R (CsTc021491)* | 5’ GAGTTCGCACTGCTTGATTGT 3’  5’ TGTGCAGCCTACAAGCCATT 3’ |  |
| *CsbHLHbHLH-F*  *CsbHLHbHLH-R (CsTc010602)* | 5’ TGCTTGATTAGGAAGCACAACA 3’  5’ GGCGGGGTACTCAGTTCAAA 3’ |  |
| *CsMyb-F*  *CsMyb-R (CsTc051689)* | 5’ TCTCCCATCCCATTGTCCCA 3’  5’ GCCCATGTAGTGAAGGAGGG 3’ |  |
| *CsSBP-F*  *CsSBP-R (CsTc051968)* | 5’ AGCCGATGATCACGGAACAA 3’  5’ ATTCTCAGGCTTCCGGCAAT 3’ |  |
| *CsSNF2-F*  *CsSNF2-R (CsTc020559)* | 5’ TTGGTCTCATGAGTCGAGCC 3’  5’ ATTTCCAGGTTGCTCGAGGG 3’ |  |
| *CsWRKY-F*  *CsWRKY-R (CsTc00677)* | 5’ TAGAGCCATCATGCACGCAG 3’  5’ ACCCATTTTGCACCTCAACC 3’ |  |
